# Inclusion of mobile phone usage guidelines in universal hypertension management protocol: an opinion

**DOI:** 10.3389/fcvm.2024.1367167

**Published:** 2024-05-30

**Authors:** Sudip Bhattacharya, Ashoo Grover, Suneela Garg, Sheikh Mohd Saleem, Abhijit Boratne, Vanita Lal

**Affiliations:** ^1^ Department of Community and Family Medicine, All India Institute of Medical Sciences, Deoghar (AIIMS Deoghar), Deoghar, India; ^2^ Implementation Research Division, Indian Council of Medical Research (ICMR), New Delhi, India; ^3^ Chair Program Advisory Committee, National Institute of Health and Family Welfare, University of Delhi, New Delhi, India; ^4^ Independent Consultant, New Delhi, India; ^5^ Department of Biochemistry, All India Institute of Medical Sciences, Deoghar (AIIMS Deoghar), Deoghar, India

**Keywords:** hypertension, blood pressure, mobile phone, NCD (non-communicable disease), epidemiologic transition, diabetes, hypertension

## Burden of hypertension

According to a global analysis published in *The Lancet* in 2019, the estimated global prevalence of hypertension among adults was approximately 1.13 billion, or 26.1% of the population aged 20 years and older (1). This prevalence has increased from 594 million in 1975, indicating a substantial rise over the years (1). Hypertension is a leading risk factor for cardiovascular diseases, including heart disease and stroke. The Global Burden of Disease study estimated that high blood pressure (BP) was responsible for approximately 10.8 million deaths worldwide in 2019, accounting for 19.4% of all deaths. Disability-adjusted life years (DALYs) represent the burden of disease, combining the years of life lost due to premature mortality and the years lived with disability. In 2019, hypertension was responsible for 182.6 million DALYs globally, indicating a significant impact on disability and overall health (2).

The burden of hypertension in India is a significant public health concern due to its high prevalence and association with cardiovascular diseases. According to a large-scale national study, the India State-Level Disease Burden Initiative, published in *The Lancet Global Health* in 2018, the prevalence of hypertension among adults in India was estimated to be approximately 29.8%. This study reported that approximately 199.5 million individuals in India were affected by hypertension (3). Hypertension-related mortality contributes to a substantial burden in India. According to the Global Burden of Disease study, in 2019, high blood pressure was responsible for approximately 1.6 million deaths in India, accounting for 14.1% of total deaths (4). Hypertension also has a significant impact on disability and overall health in India. The India State-Level Disease Burden Initiative estimated that hypertension accounted for 2.4 million DALYs in India in 2017, representing a considerable burden of disease (5). A study published in 2020 examined the risk factors associated with hypertension in India. It found that factors such as older age, urban residence, higher body mass index (BMI), and diabetes were significantly associated with hypertension (3). Another study also highlighted the low awareness and control rates of hypertension in India, emphasizing the need for improved screening and management (6).

Blood pressure is typically checked using a device called a sphygmomanometer. There are two main types of blood pressure measurements: manual (using an aneroid or mercury sphygmomanometer) and automated (using an electronic or digital device).

As per standard guidelines, the person having their blood pressure checked should be seated comfortably in a quiet environment; the guidelines also recommend refraining from talking as talking may include the use of mobile phones for a few minutes before the blood pressure measurement. The individual should avoid smoking, caffeine, and exercise for at least 30 min before the blood pressure measurement (7).

## Problem statement

It is not uncommon for patients to use their mobile phones to surf the Internet while waiting in queues, including those at doctor's offices. Mobile devices provide easy access to various forms of entertainment, information, and communication, making them a convenient way to pass the time. The use of social media and mobile devices has become pervasive in today's society, and although they offer many benefits, they can also contribute to mental health disorders such as stress and anxiety in individuals. A study identified different profiles of social media addiction among college students and found a positive association between social media addiction and symptoms of anxiety (8).

Another study revealed that problematic social media use was associated with poor sleep quality, attention-deficit hyperactivity disorder (ADHD), and lower self-esteem among adolescents (9). A study on a similar topic analyzed online information related to anxiety and depression and found that the quality of information on social media platforms was variable, and inaccurate or misleading content could contribute to increased stress and anxiety (10). Another study explored the relationship between digital technology use and wellbeing in adolescents and found that the association between digital technology use and mental health outcomes, including stress and anxiety, was small but statistically significant (11). To our knowledge, the association between increase in mobile phone usage and increase in BP is very scarce, and the available evidence is indirect, which is commonly interacted with mental disorders such as stress and anxiety.

A study involved participants with a mean age of 54 years, consisting of 62% women and 88% mobile phone users. Over a median follow-up period of 12 years, 13,984 (7%) participants developed hypertension (12). Mobile phone users had a 7% increased risk of developing hypertension compared to non-users. Among mobile phone users, those who spent 30 min or more per week on phone calls had a 12% higher likelihood of developing high blood pressure compared to participants who spent less than 30 min on calls. These associations were observed in both women and men. Further analysis revealed more detailed findings regarding the relationship between weekly usage time and the risk of high blood pressure. Compared to participants who spent less than 5 min per week on phone calls, those who spent 30–59 min, 1–3 h, 4–6 h, and more than 6 h had an 8%, 13%, 16%, and 25% higher risk of developing hypertension, respectively (9). However, among mobile phone users, the number of years of use and the use of hands-free devices or speakerphones did not significantly affect the likelihood of developing hypertension. The researchers also investigated the impact of usage time (less than 30 vs. 30 min or more) on new-onset hypertension in relation to participants’ genetic risk levels for developing hypertension. Genetic risk was determined using data from the UK Biobank. The analysis demonstrated that individuals with a high genetic risk who spent at least 30 min per week on mobile phone calls had a 33% higher likelihood of developing hypertension compared to those with a low genetic risk who spent less than 30 min per week on the phone (13).

In this study, a significant decreasing trend was found between systolic blood pressure (SBP), diastolic blood pressure (DBP), and heart rate and higher mobile phone usage in women. Based on a regression analysis, SBP, DBP, and duration of mobile phone use were associated negatively in those who used their phones for at least 8 h, which is in contrast with our previous study. The type of use and content may determine the impact on blood pressure. For example, if someone is scrolling funny videos, their BP may decrease; on the other hand, if a person is constantly looking at the stock market, their BP may be on the higher side (14).

In our opinion, using a mobile phone before a blood pressure checkup can potentially influence the blood pressure reading. This phenomenon may be called a “confounding effect in hypertension” or “mobile phone error in hypertension measurement,” where a person's blood pressure may be higher when measured in a clinical setting due to anxiety or stress associated with the medical environment or mobile phone-induced stress.

Few studies have explored the impact of mobile phone use on blood pressure measurements. For example, the “European Society of Hypertension guidelines for blood pressure monitoring at home: a summary report of the Second International Consensus Conference on Home Blood Pressure Monitoring” mention that activities such as smoking, exercise, and using a mobile phone should be avoided for at least 30 min before blood pressure measurements (15). Still, this guideline is yet to be widely accepted and implemented. Another study investigated the acute effects of exposure to mobile phone electromagnetic fields on blood pressure and found that it had a small but significant effect on increasing blood pressure measurements (16).

The exact mechanisms underlying how using a mobile phone before a blood pressure checkup may influence blood pressure readings are not yet fully understood. Several factors associated with mobile phone use can impact blood pressure measurements. First, engaging in activities such as checking work emails or receiving distressing news on a mobile phone can induce psychological stress and anxiety, both recognized as temporary elevators of blood pressure levels. In addition, the act of using a mobile phone itself can lead to distraction, diverting attention from relaxation—an essential state for accurate blood pressure readings. Poor posture during mobile phone use, characterized by bending the neck and shoulders forward, can induce temporary changes in blood flow dynamics and blood pressure regulation, potentially affecting measurements. Moreover, some studies suggest that exposure to electromagnetic fields emitted by mobile phones may directly influence the autonomic nervous system, responsible for blood pressure regulation. However, the evidence supporting this specific physiological arousal mechanism is currently limited and inconclusive.

It is important to note that the impact of mobile phone use on blood pressure measurements may vary between individuals and depend on factors such as their baseline blood pressure, level of stress, and the duration and intensity of mobile phone use.

## Recommendations

Future research in the area of mobile phone use and its influence on blood pressure measurements could explore the following avenues: conducting long-term studies that follow individuals over an extended period could provide valuable insights into the long-term effects of mobile phone use on blood pressure. This would help determine whether any observed influences are transient or persistent. In addition, using experimental designs, researchers can manipulate variables such as mobile phone use duration, content type, and posture to better understand their impact on blood pressure. Controlled experiments can provide more precise insights into the causal relationships between mobile phone use and blood pressure changes. We can also investigate whether studies on the potential effects of mobile phone electromagnetic field exposure on blood pressure regulation. Research could involve assessing physiological responses to electromagnetic field exposure and exploring potential mechanisms through which it might influence blood pressure. Studying the effectiveness of smartphone applications or interventions specifically designed to mitigate the potential negative effects of mobile phone use on blood pressure could be another interesting area of research. This could involve developing and testing apps that promote relaxation, stress reduction, and proper posture during mobile phone use. Investigating individual factors that may modulate the impact of mobile phone use on blood pressure could be valuable. Factors such as age, baseline blood pressure, stress levels, and psychological factors could be considered to better understand why some individuals may be more susceptible to blood pressure changes due to mobile phone use. Operational research conducted in healthcare clinics or workplaces could provide insights into the practical implications of mobile phone use on blood pressure measurements. Understanding the influence of environmental factors and contextual variables on the relationship between mobile phone use and blood pressure would be important. By exploring these research avenues, we can enhance our understanding of the complex relationship between mobile phone use and blood pressure and develop evidence-based guidelines for restricting mobile phone usage before measuring accurate BP cases in various settings.
